# Microfilaria persistent foci during post MDA and the risk assessment of resurgence in India

**DOI:** 10.1186/s41182-018-0107-8

**Published:** 2018-07-17

**Authors:** Pramod Kumar Mehta, Ramanuj Rauniyar, Birendra Prasad Gupta

**Affiliations:** 1 Vector Control Research Centre, Medical Complex, Indira Nagar, Pondicherry, India; 2 Kathmandu Research Institute for Biological Sciences, Kathmandu, Nepal; 3 Central Diagnostic Laboratory and Research Center Pvt. Ltd, Kathmandu, Nepal; 4 Present address: Central Regional Health Directorate Office, Ministry of Health and Population, Kathmandu, Nepal

**Keywords:** Lymphatic filariasis, *Wuchereria bancrofti*, Mass drug administration

## Abstract

**Background:**

Pondicherry, *a union territory in India*, is an endemic district for bancroftian lymphatic filariasis transmitted by *Culex quinquefasciatus* where eight rounds of mass drug administration (MDA) were completed in 2011 (annually once from 2004 to 2011).The objectives of this study were to conduct a focal survey to assess microfilaria and antigen (Ag) prevalence among young adults and to assess vector infection and infectivity through a focal entomological survey.

**Methods:**

Mosquitoes were collected using gravid traps in Sedurapet village of Pondicherry and dissected to enumerate *W. bancrofti* larvae stage first larval stage (L1), second larval stage (L2), and third larval stage (L3). Microfilarias (Mf) were detected using blood smears collected from inhabitants.

**Results:**

A total of 360 individuals from 67 houses were enrolled in this study of which 290 (80.6%) were surveyed for the presence of Mf. Two Mf carriers were detected yielding an overall prevalence of 0.69% and two out of 85 (2.35%) were Mf antigen positive. Of the 2875 mosquitoes collected by gravid trap, *Culex quinquefasciatus* (93.9%) was the predominant species, followed by *Anopheles subpictus* (2.3%) and *Culex vishnui* (3.8%). The density of *Cx. quinquefasciatus* was 28.1 per trap-night. A total of 2429 *Cx. quinquefasciatus* were dissected and microscopically examined for abdominal conditions (gravid 85%, semi-gravid 9.4%, unfed 3.8%, and fully fed 1.9%) and filarial infection. One mosquito (infection rate equal to 0.04%) was found to harbor a second stage filarial larva, and none of the mosquitoes had infective stage larva.

**Conclusion:**

Our results show no reappearance of infection of lymphatic filariasis in Sedurapet village of Pondicherry after MDA, and thus, no further intervention is required in that area for possible resurgence of lymphatic filariasis. However, monitoring should be continued as part of post MDA activities until the endpoint of complete elimination is achieved. We demonstrated that xenomonitoring can be used to monitor the post MDA situation for possible risk of transmission to initiate control measures.

## Background

Lymphatic filariasis is targeted for global elimination by 2020 [1]. Preventive chemotherapy using a mass annual single dose of diethylcarbamazine (DEC), and albendazole is the main transmission control strategy in preventing new infections and achieving elimination [2]. All the three filarial parasites viz., *Wuchereria bancrofti*, *Brugia malayi*, and *Brugia timori* with their physiological races of periodic and subperiodic, are known to respond well to the drugs used in the program [3]. The Programme to Eliminate Lymphatic Filariasis (PELF) was launched in 2000 following the resolution of World Health Assembly 2007 [1]. Thirteen countries have successfully interrupted the transmission and are under post MDA surveillance [4]. However, global elimination by 2020 remains a challenge with seven countries yet to start MDA, although mapping has already been completed [5].

As per the WHO recommendations, post MDA surveillance has to be carried out for 5 years after stopping MDA based on the evidence of absence of transmission [6]. The post MDA surveillance tools and indicators should be valid and sensitive enough to indicate the possible risk of resurgence [7]. Preparedness to prevent or control such reappearance of infection is therefore necessary. The absence of transmission can be an appropriate indicator, which is defined as reducing transmission of the parasite to a level (antigen prevalence less than 1% in 6–7 years of children) where continued transmission and recrudescence are not expected [8]. The present study aimed to assess the status of transmission in communities with persistent infection (residual Mf carriers) (Table 1).

**Table 1:** Summary of infection and transmission parameters

Parameters	Total	Percentage
Total number of population of Sedurapet village	4433	
Number of house selected for focal survey	67	
Enumerated population from the selected houses	360	
Number of individuals covered for MF screening	290/4433	6.5
Number of Mf positive cases detected	2/290	0.6
Number of individuals covered under antigenemia screening	85/4433	1.9
Number showing positive reaction to antigen test	2/85	2.3
Number of children below 5 years	17/360	4.7
Total number of mosquitoes trapped from gravid traps	2875	
Number of *Cx. quinquefasciatus* recorded	2700/2875	93.9
Number of *Cx. quinquefasciatus* dissected	2429/2700	89.9
Vector infection rate	1L2larvae/2429	0.04
Other mosquitoes	175/2875	6.1
Per trap density of *Cx. quinquefasciatus*	96 trap/16 days	28.1

## Method

### Study area

The Sedurapet village in Pondicherry, *a union territory in India*, was selected for the study. It lies between 11.45 to 12.15^0^N latitude north and 79.35 to 80.00°E longitude east, on the Coromandel coast of peninsular India. The mean monthly temperature of Sedurapet village ranges between 30.8 °C in May and 25.1 °C in January. The population of this village was 4433 and endemic for bancroftian filariasis. The human dwellings of the village are of three types, viz., thatched huts, houses with tiled roofs, and terraced houses. Stagnant drains, numerous cesspits, cesspools, vacant plots, septic tanks, cement tanks, and water collection near public taps provide highly conducive breeding places for *Culex quinquefasciatus*. The area was covered under MDA from 2004, and eight rounds of MDA were completed by 2011.

### Selection of house and parameters used in the study

This study was carried out only in 67 selected houses. All 67 HH (household) populations were involved in the study. The following parameters were used in this study:

- Mf prevalence = total no. of smear-positive for Mf × 100/total no. of smear examined for Mf
  = 2 × 100/290
  = 0.69%
  Ag prevalence = total no. of Ag positive cases × 100/total no. cases examined
- for Ag
- Vector infection rate = total no. of L1 or L2 stage of *W. bancrofti* × 100/total no. of gravid mosquitoes dissected
  = 1 × 100/2429
  = 0.04%
- Vector infectivity rate = total no. of L3 stage of *W. bancrofti* × 100/total no. of gravid mosquitoes dissected
  = 0 × 100/2429
  = 0%

### Night blood survey for Mf prevalence

**Fig. 1:**
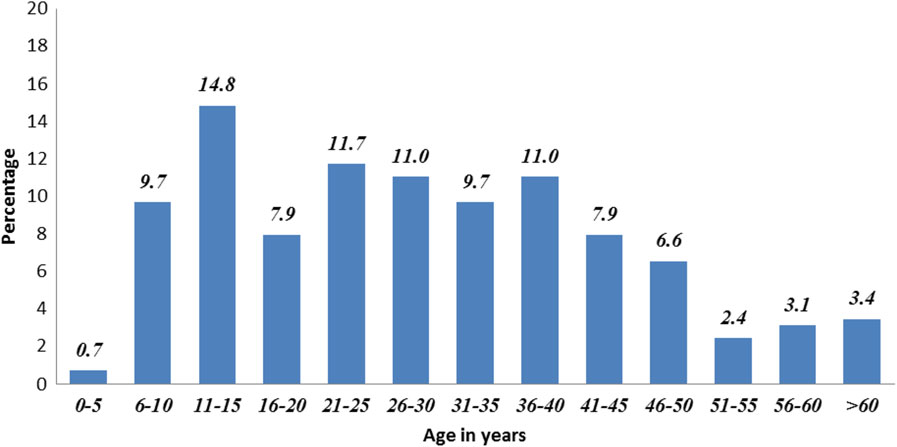
Percentage of Mf survey population in each age class

As the study area was endemic for filariasis caused by a nocturnally periodic form of *Wuchereria bancrofti*, the blood samples from all the available individuals were collected at night between 10.00 PM to midnight. Written consent was obtained from all individuals and from parents of children below the age of 15 years. A team of four technical staff, one for registering names, another for preparing slides, a third for drawing blood samples, and a fourth for making smears were involved. One hundred microliters of blood were smeared on slides and marked in the center with a lead pencil. Dehaemoglobinization was done for about 3 min in tap water. After air drying, the blood films were fixed in acid-alcohol (2 parts of concentrated HCl + 98 parts of methyl alcohol), stained in Jaswant-Singh-Bhattacharji stain (JSB) for 1–2 min, and washed in distilled water. After subsequent air drying, the blood smear was examined under a compound microscope using X10 objective (Fig. 1).

### Antigenemia survey

While collecting blood samples for microfilaria detection, children and young adults (6–25 years) were also tested for the presence of *W. bancrofti* antigen in all 67 households. The purpose of the survey was to verify whether there was any evidence for recent transmission as well as to determine the prevalence of infection among young adults. The ICT (Binax, Portland, ME) was used for the detection of *W. bancrofti* antigen in a whole blood sample (Table 2).

**Table 2:** Age-wise summary of antigen survey

Age group	Number antigen positive out of (*n = 85)* in each age group	Percentage
6–10	26	30.5
11–15	23	27.0
16–20	14	16.4
21–25	22	25.8

### Vector sampling

Six houses in four streets (Mettu Street, Kurinji Street, Mohambigai Street, and North Street) of the Sedurapet village were selected, and gravid traps were placed for collecting vector mosquitoes. Method of collecting vector mosquitoes using gravid traps was standardized in the field with respect to the site of placement, appropriate time, and attractiveness of infusion. Six gravid traps were kept in 96 places covering all 67 HH for the collection of mosquitoes for 16 days. All collected mosquitoes were dissected for the study of infection and infectivity.

### Trapping procedure

The traps were placed for 16 days in the evening and the collection bag was removed the following morning and sent to the laboratory for further analysis. Trapped mosquitoes were removed by using a mechanical aspirator, and female *Cx. quinquefasciatus* mosquitoes were identified. These were later separated into test tubes labeled with trap number and date of collection.

### Assessment of vector infection

**Fig. 2:**
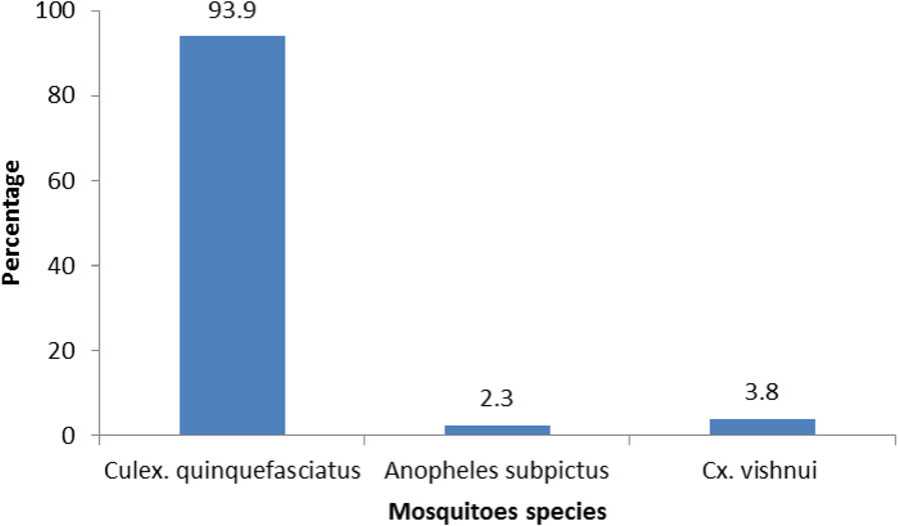
Mosquitoes species composition of gravid trap collection

Mosquitoes collected from the gravid traps were dissected on the same day. Filarial larvae detected in the infected mosquitoes were categorized into microfilariae (Mf), stage I (L1), II stage (L2), and III stage (L3) infective stage as per the classification of Samarawickrema et al. 1967 [9]. Mosquitoes carrying any stage of the filarial parasite (Mf, L1, L2, or L3 larva) were defined as “infected,” while those carrying only L3 larva were defined as “infective.” The number of each stage of larvae in the infected mosquitoes was counted and recorded (Fig. 2).

### Statistical analysis

Exploratory data analysis was carried out using Microsoft Excel software. The prevalence of antigenemia or prevalence of Mf was calculated as the number of persons positive for Ag or Mf divided by the number tested.

## Results

### Microfilaria survey

The present survey was carried out 24 months after the last MDA. A total of 360 people were enumerated in 67 households in the study area. Out of 360 enlisted individuals, 290 (80.6%). were included in the Mf survey (Fig. 1). As the target population was above 5 years of age, the coverage in different age groups ranged between 64 and 100% and about 90% coverage was achieved among children between 6 and 15 years. Only two Mf carriers (aged 45 and 60 years) were detected yielding an overall prevalence of 0.69%. The microfilaria density was 5 per 60 μl.

### Antigenemia survey

**Fig. 3:**
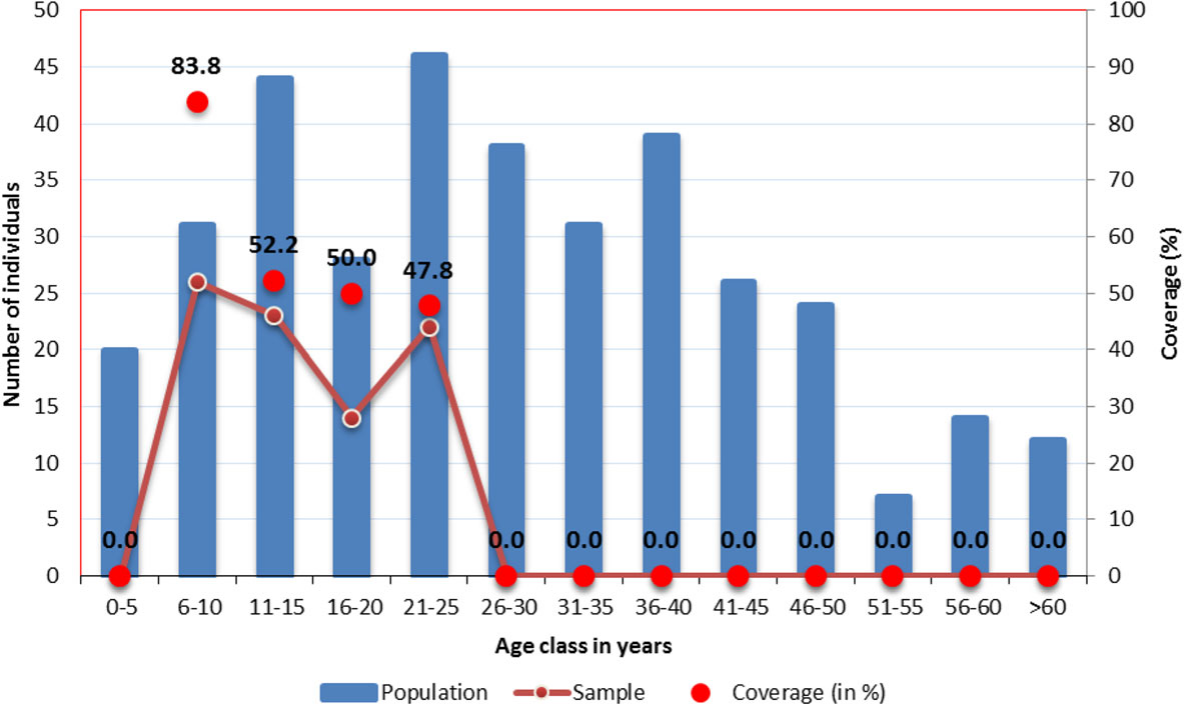
Distribution of population and sample for antigen survey and coverage on different age group

Two (2.35%) individuals out of 85 aged between 5 and 25 years were positive for antigenemia (Fig. 3).

### Entomological survey

A total of 2875 mosquitoes from six houses were trapped. *Cx. uinquefasciatus* (93.6%) was the predominant species recorded from the gravid trap (Fig. 2) followed by *Anopheles subpictus* and *Cx. Vishnui*. The average density of *Cx. quinquefasciatus* was 28.1 per trap per night. Two thousand four hundred twenty-nine female mosquitoes were dissected for filarial infection and based on abdominal condition, gravid mosquitoes constituted about 85%, followed by semi-gravid (9.4%), unfed (3.8%), and fully fed (1.9%). One mosquito was found to harbor a second stage filarial larva yielding an infection rate of 0.04%. None of the mosquitoes had infective stage larva.

## Discussion

When the Annual Mass Drug Administration Programme to eliminate LF was originally launched, guidelines for program planning, implementation, monitoring, and evaluation were available [10]. However, tools and protocols for monitoring and evaluation were not operationally feasible with highly conservative threshold levels. Compliance has been a major operational issue identified as a limiting factor for effective implementation of MDA. In India, a number of independent assessments of program coverage have been carried out [11]; however, issues identified were stated to be situation specific, requiring site-specific measures to bridge the gap between coverage and compliance. The minimum effective coverage of the total population was estimated to be 65% [10]. Even though five rounds of MDA were recommended as the minimum target, a systematic review on mass chemotherapy options to control LF [10] and other studies have shown that the required number of rounds of MDA depends on several factors including initial prevalence of infection, initial intensity of transmission, efficacy of drugs, the combination of parasites and vectors, and the density of vectors [12].

The existence of foci of persistent infection and transmission has also been reported. This is defined as the prevalence of infection (Mf and or antigenemia) above 1% or prevalence of antigenemia in children between 2 and 8 years [13]. These areas require particular attention during post MDA surveillance in order to detect any signals for a possible resurgence of infection. The effect of long-term observations on the status of such areas is still unknown. Two Mf carriers detected in a previous survey were both identified to be migrants; however, the two identified in the current study did not indicate any history of the movement to the endemic areas and hence were considered as indigenous cases. The persistence of infection in terms of Mf carriers was thus evident. The present focal survey detected the indigenous cases, and the migrants had already left and were not available at the time of the second survey. Screening of children and young adults also showed 2.35% antigenemia prevalence. This trend is as expected since antigen rates decrease more slowly than Mf rates.

The detection of the filarial parasite in the vector mosquito is an indication of the presence of Mf carriers in the sampled location. The mosquito recorded with filarial parasite infection in the study was found in a house where none of the members were Mf carriers. This indicates that vector sampling from the location could detect filarial infection and thus can be a proxy indicator of infection in the sampling area. This can thereby be an appropriate tool to indirectly assess the prevalence of infection. Passive vector sampling can be a potential surveillance tool in place of night blood surveys, which require larger sample size in situations of low Mf prevalence, which can be expected in areas under preventive chemotherapy.

The current study area is an industrial area with a large number of migrant laborers from other states; therefore, this area is in demand of strengthened monitoring. Testing and treating high-risk populations such as migrants can be a potential option and vector control could be yet another solution to ensure that recrudescence will not occur from residual Mf carriers. However, limitations in carrying out post MDA surveillance activities are constrained by limited manpower. Therefore, integrating post MDA surveillance activities with those of other neglected tropical diseases (NTDs) control program or integrating LF surveillance activity with population-based surveys could reduce the need for long-term resources for LF specific surveillance [14].

The present study showed that gravid traps could serve as an effective surveillance tool to sample vector mosquitoes as it yields 25 mosquitoes per trap per night. In places where adequate resources are available with facilities for molecular-based detection of filarial infection, pooled mosquito samples can be processed for detecting filarial infection including infectivity for which tools and protocols have already been developed [15]. Mass distribution of DEC salt could be an alternative intervention in the transmission hot spots to prevent recrudescence [16].

## Conclusions

In this study, there was no active transmission in the sampled area (Mf prevalence < 1%) during the post MDA period. Consequently, in this region, no further intervention is required to avoid a resurgence of lymphatic filariasis. However, monitoring should be continued as a part of post MDA activities until the ultimate endpoint of elimination is achieved (< 1% Mf). Xenomonitoring could be a potential tool for this allowing control measures to be initiated in a timely manner should resurgence be detected [17–20].

## Abbreviations

DEC: Diethylcarbamazine
ICT: Immunochromatic card test
LF: Lymphatic filariasis
MDA: Mass drug administration
Mf: Microfilaria
NTDs: Neglected tropical diseases
PDA: Personal digital assistant
PELF: Programme to Eliminate Lymphatic Filariasis
VCRC: Vector Control Research Centre
WHO: World Health Organization
